# Understanding implementation fidelity in a pragmatic randomized clinical trial in the nursing home setting:a mixed-methods examination

**DOI:** 10.1186/s13063-019-3725-5

**Published:** 2019-11-28

**Authors:** Jennifer A. Palmer, Victoria A. Parker, Lacey R. Barre, Vincent Mor, Angelo E. Volandes, Emmanuelle Belanger, Lacey Loomer, Ellen McCreedy, Susan L. Mitchell

**Affiliations:** 1 Harvard Medical School, 25 Shattuck Street, Boston, MA 02215 USA; 2 Hebrew SeniorLife, Hinda & Arthur Marcus Institute for Aging Research, 1200 Centre Street, Roslindale, MA 02131 USA; 3 Department of Medicine, Beth Israel Deaconess Medical Center, East Campus, Yamins 419, 330 Brookline Avenue, Boston, MA 02215 USA; 4 Peter T. Paul College of Business and Economics, University of New Hampshire, 10 Garrison Avenue, Durham, NH 03824 USA; 5 Department of Health Services, Policy, and Practice, School of Public Health, Brown University, 121 S Main St, Providence, RI 02903 USA; 6 Center for Gerontology and Healthcare Research, School of Public Health, Brown University, 121 S Main St, Providence, RI 02903 USA; 7 Center of Innovation in Health Services Research and Development Service, Providence Veterans Administration Medical Center, 830 Chalkstone Ave, Providence, RI 02908 USA; 8 Section of General Medicine, Massachusetts General Hospital, 55 Fruit Street Gray 7-730, Boston, MA 02114 USA

**Keywords:** Pragmatic trial, Implementation, Fidelity, Nursing homes, Mixed methods

## Abstract

**Background:**

The Pragmatic Trial of Video Education in Nursing Homes (PROVEN) is one of the first large pragmatic randomized clinical trials (pRCTs) to be conducted in U.S. nursing homes (*N* = 119 intervention and *N* = 241 control across two health-care systems). The trial aims to evaluate the effectiveness of a suite of videos to improve advance care planning (ACP) for nursing home patients. This report uses mixed methods to explore the optimal and suboptimal conditions necessary for implementation fidelity within pRCTs in nursing homes.

**Methods:**

PROVEN’s protocol required designated facility champions to offer an ACP video to long-stay patients every 6 months during the 18-month implementation period. Champions completed a video status report, stored within electronic medical records, each time a video was offered. Data from the report were used to derive each facility’s adherence rate (i.e., cumulative video offer). Qualitative interviews held after 15 months with champions were purposively sampled from facilities within the highest and lowest adherence rates (i.e., those in the top and bottom quintiles). Two researchers analyzed interview data thematically using a deductive approach based upon six domains of the revised Conceptual Framework for Implementation Fidelity (CFIF). Matrices were developed to compare coded narratives by domain across facility adherence status.

**Results:**

In total, 28 interviews involving 33 champions were analyzed. Different patterns were observed across high- versus low-adherence facilities for five CFIF domains. In low-adherence nursing homes, (1) there were limited implementation resources (Context), (2) there was often a perceived negative patient or family responsiveness to the program (Participant Responsiveness), and (3) champions were reticent in offering the videos (Recruitment). In high-adherence nursing homes, (1) there was more perceived patient and family willingness to engage in the program (Participant Responsiveness), (2) champions supplemented the video with ACP conversations (Quality of Delivery), (3) there were strategic approaches to recruitment (Recruitment), and (4) champions appreciated external facilitation (Strategies to Facilitate Implementation).

**Conclusions:**

Critical lessons for implementing pRCTs in nursing homes emerged from this report: (1) flexible fidelity is important (i.e., delivering core elements of an intervention while permitting the adaptation of non-core elements), (2) reciprocal facilitation is vital (i.e., early and ongoing stakeholder engagement in research design and, reciprocally, researchers’ and organizational leaders’ ongoing support of the implementation), and (3) organizational and champion readiness should be formally assessed early and throughout implementation to facilitate remediation.

**Trial registration:**

ClinicalTrials.gov, NCT02612688. Registered on 19 November 2015.

## Background

Pragmatic randomized clinical trials (pRCTs) are increasingly employed to maximize the translation of evidence-based interventions into practice. These trials differ from explanatory (i.e., traditional) RCTs in design and intent. As conceptualized by the Pragmatic Explanatory Continuum Indicator Summary version 2 (PRECIS-2) framework [1], trials can fall along a pragmatic to explanatory continuum in various design features. Traditional RCTs emulate highly controlled conditions to evaluate an intervention’s efficacy, whereas pRCTs emulate real-world conditions to evaluate an intervention’s effectiveness. Based on the National Institute on Aging’s Stage Model for Behavioral Intervention Development [2], pRCTs are typically conducted at the later stages in evaluating an intervention.

Implementation fidelity is important for accurately interpreting findings of both efficacy and effectiveness trials, but are a particular concern for pRCTs, in which, by design, adherence to the intervention is less tightly controlled by the research team. The importance of fidelity in traditional RCTs has long been recognized, and the National Institutes of Health Behavior Change Consortium has recommended practices for ensuring and assessing fidelity within this context [3]. While there is growing recognition of the importance of and challenges to maintaining fidelity in pRCTs [4, 5], similar consensus recommendations have not yet been published. Moreover, while factors influencing implementation fidelity have been studied in traditional RCTs, research into these factors in pRCTs is nascent [4, 6].

Mixed-methods study designs are a valuable approach in deciphering the why and how of success or failure in achieving fidelity within pRCTs [7]. A qualitative analysis can unearth the influence of context and setting and contribute to the interpretation of quantitative results related to intervention fidelity. The integration of these research methods can identify strategies to ameliorate or avoid situations where an intervention’s core elements are not delivered as intended or emulate situations where they are.

This report aims to use mixed methods to further our understanding of factors influencing fidelity to a trial protocol within the context of pRCTs by leveraging data from the Pragmatic Trial of Video Education in Nursing Homes (PROVEN), which is one of the largest pRCTs to be conducted in nursing homes. PROVEN implements an advance care planning (ACP) video education program in intervention facilities. ACP, the process by which clinicians determine patients’ and families’ preferred treatment decisions prior to treatment needs, most appropriately results in advance directives (e.g., do not hospitalize or do not resuscitate). Nursing homes are required to engage in this process; however, evidence reveals deficiencies in meeting this mandate [8–17]. Video decision-support tools have been developed to standardize information about and provide visualization of treatment decisions and to obviate literacy and language barriers inherent to traditional ACP. While such tools have resulted in improvements to ACP in small RCTs across a number of settings [18–25], pRCTs could substantiate the benefit of the tools’ broad uptake in real-world settings including nursing homes. Using modified Conceptual Framework of Implementation Fidelity (CFIF) domains [26], we used a deductive qualitative analysis to compare the experiences of champions in facilities with low versus high quantitative adherence rates to explore how to optimize implementation fidelity within pRCTs in nursing homes.

## Methods

### Overview of PROVEN

Brown University’s Institutional Review Board gave approval to PROVEN and determined that the nursing home providers were not engaged in research using human subjects. Extensive detail on the trial design can be found elsewhere [27].

In brief, PROVEN was conducted in two large nursing home health-care systems in the U.S. (total *N* = 360 nursing home facilities; health-care system 1: *N* = 98 intervention, *N* = 199 control; health-care system 2: *N* = 21 intervention, *N* = 42 control). The trial began in March 2016 and was completed in May 2019. The intervention included a suite of five videos (~ 6–10 min in length) to aid in health-care decision-making: (1) General Goals of Care, (2) Goals of Care for Advanced Dementia, (3) Hospice, (4) Hospitalization, and (5) Advance Care Planning for Healthy Patients. These videos were loaded onto tablet devices, and two tablets were provided to each intervention facility. Family members could also access the videos online on their own devices through a password-protected internet link.

The core components of the intervention included (1) offering the video to patients and families and (2) doing so within specified time parameters. At each intervention facility, one or two ACP champions (most often a social worker) were designated as the individuals responsible for delivering the intervention. As per the implementation protocol, they were instructed to offer a video to all newly admitted or readmitted patients (or their family members) within 7 days of admission and to all long-stay patients (length of stay > 100 days) every 6 months or upon change in status over an 18-month implementation period. Consistent with a pragmatic trial, other elements of the program were customizable to real-world demands (e.g., which videos were offered, to whom they were offered, and which mode of administration was offered or used). We enclose the mplementation guides for champions (Additional file 1) and for intervention facilities (Additional file 2).  ACP practices continued as per usual in the control facilities.

While the study population included all patients in the nursing home during the implementation period, for analytic purposes we identified a target cohort, using Minimum Data Set assessments, of long-stay residents with advanced dementia, chronic obstructive lung disease, or heart failure. The primary trial outcome was the difference in hospital transfer rates per person-day alive over 12 months in this advanced illness cohort between the intervention and control arms.

The research team and health-care system leadership partnered in designing the PROVEN protocol and in training ACP champions. In contrast to a traditional RCT, the ACP video program roll-out and ongoing implementation were primarily led by the health-care system corporate leadership, that is, in a fashion that was typical for any new quality improvement program being introduced into their systems. The health-care system leaders, but not the ACP champions, staff, patient, or families, were aware that the ACP video program was being tested in a RCT.

#### Measuring adherence

To assess adherence to the protocol, the research team designed a short video status report that was embedded in the electronic medical record system at all intervention nursing homes. Each time a champion offered a video to a patient or family member, they were instructed to complete the video status report, which included closed questions on the date the video was offered, whether it was viewed (e.g., the patient or family may have refused to watch it), and if shown, which video was shown and to whom. The research team was able to link the video status reports to the Minimum Data Set data to determine the proportion of new admissions and long-stay residents who had a completed video status report, implying a video was offered, as per the protocol. During the implementation period, the health-care system leadership gave the champions monthly feedback reports, which included their facility’s adherence or video offer rate. These reports were reviewed at regular telephone group and individual conference calls with health-care system leadership or research team members.

To be consistent with PROVEN’s focus on long-stay patients within its primary trial outcome, adherence in this report was measured using the cumulative completion rates of video status reports for long-stay patients only (to the exclusion of rates for short-stay patients). This rate was calculated across each facility in the health-care systems that had consistently collected interview data. Nursing homes were categorized into quintiles based on the distribution. Nursing homes in the top quintile were considered high-adherence facilities and those in the bottom, low-adherence facilities.

#### Semi-structured champion interviews

An experienced research assistant conducted semi-structured telephone interviews with champions from all intervention facilities to gather their perceptions of the implementation at 4 months, 9 months, and 15 months into the implementation. Qualitative data for this report were derived from the 15-month interviews (Additional file 3), which included questions on: (1) the overall implementation experience (e.g., strengths; weaknesses; and patient, family, and non-champion staff reactions), (2) the champion’s efforts at facilitating program implementation, (3) how the program may have changed the champion’s ACP conversations and practices, (4) the champion’s impression of whether program implementation became easier or harder over time, (5) suggestions for improving the program, and (6) the champion’s proclivity to recommend the intervention to other nursing homes. Interviews were recorded and professionally transcribed.

### Conceptual framework

We based our deductive analysis on CFIF given its establised use in the literature and its multi-faceted approach to understanding implementation fidelity. According to CFIF [28], implementation fidelity is a multi-component construct that represents adherence (the bottom-line measurement of fidelity) and associated moderators. Adherence includes the following components: (1) content (the intervention’s active ingredients), (2) frequency (how often the active ingredients were delivered), (3) duration (for how long the active ingredients were delivered), and (4) coverage (how much of the active ingredients were delivered). Given the intervention’s design, only frequency (i.e., the cumulative completion rate of video status reports, which reflected whether each video was offered to each patient as per the protocol) is relevant to this report. Duration and coverage were considered to be too unstable to include in the adherence score given their potential variation across patients and family members.

The original CFIF uses four constructs (also called domains) that moderate the relationship between program implementation and adherence: (1) Intervention Complexity, (2) Participant Responsiveness, (3) Quality of Delivery, and (4) Strategies to Facilitate Implementation [28]. It is proposed that interventions that are less complex in structure are easier to implement with high fidelity. Participant Responsiveness considers both those participants delivering and those receiving an intervention. Within PROVEN, “participants” refers to champions, patients, and family members. It is assumed that if participants respond more positively to the intervention (the videos), implementation fidelity will be higher. Quality of Delivery refers to the degree to which intervention delivery aligns with its theoretically intended purpose. For PROVEN, the ACP video program was intended to supplement ACP conversations between champions and patients and family members to improve goal-directed medical decision-making. Poor delivery of the intervention content may translate into a suboptimal degree of implementation fidelity. Strategies to Facilitate Implementation are conceptualized as those activities initiated to achieve optimal and standardized implementation fidelity. In PROVEN, facilitation strategies (e.g., feedback reports) could be initiated by the research team, the health-care system leadership, or the champions.

The modified version of CFIF, which guides this report, adopts these four moderating constructs and proposes two additional ones: Recruitment and Context [26]. Recruitment encompasses the methods employed to solicit the participation of nursing home residents in the ACP video intervention, the consistency of these methods, and the reasons for non-participation by the individuals recruited. In PROVEN, such methods included efforts to solicit patient and family member participation prior to offering the video and when it was offered, up until when the video was shown. Within the modified CFIF, Context relates to the environment (the organizational structure and culture as well as co-occurring and historical events) encompassing the intervention. In PROVEN, this environment relates to each intervention facility’s structure and culture as well as, for example, coinciding programs or policies designed to lower hospital transfer rates.

#### Mixed-methods approach and analysis

This mixed-methods report followed a sequential explanatory design and integrated PROVEN’s quantitative and qualitative data at the methods level. This was done by connecting the two datasets through the sampling framework [29]. Sites were sampled and a mixed-methods analysis was conducted toward the end of the trial; thus, the results of this report were not shared with sites during the implementation.

Within each health-care system, nursing homes in the top (high-adherence) and bottom (low-adherence) quintiles of adherence rates were identified based on the video status reports. Facilities with an adherence rate of zero were excluded; these facilities were disengaged from the program for a wide variety of reasons (e.g., pending closure and administrative upheaval) to the extent that feedback from their champions would not have been informative. Only interviews from these high- and low-adherence nursing homes were analyzed. The analysis was done deductively across the two facility classifications to identify differential factors influencing fidelity as established by the six modified CFIF constructs [26]. Two researchers (JAP and LRB), blinded to facility adherence status (i.e., high or low), developed a structured codebook (Additional file 4)  and independently coded all data by blocks of text through an iterative process. The researchers actively identified instances where codes were verified or refuted within the data. Consensus meetings between JAP and LRB were periodically held to reconcile individual coding decisions. The software NVivo 11 (QSR International; Melbourne, Australia) was utilized to organize and manage the data. When the coding was complete, JAP was unblinded to facility adherence status and developed matrices to compare the coded narrative by CFIF domain across high- versus low- adherence facilities to identify any similarities and differences in implementation factors. These themes were then assessed according to the following classification: (1) paired-convergent: the theme was represented in both high- and low-adherence facilities with similar findings, (2) paired-divergent: the theme was represented in both high- and low-adherence facilities but with contrasting findings, and (3) unpaired: the theme was represented in a high- or low-adherence facility, but not both.

## Results

A total of 28 facilities from each of the two health-care systems were sampled from the top and bottom quintiles of adherence rates (Table 1). In health-care system 1, there were 11 facilities in the top quintile (range 66–92%) and 11 facilities in the bottom quintile (range 24–40%). In health-care system 2, there were 3 facilities in the top quintile (range 39–78%) and 3 facilities in the bottom quintile (range 12–24%).

**Table 1:** Adherence rates within top and bottom quintiles for PROVEN’s two health-care systems

	Health-care system 1	Health-care system 2
Top quintile	66–92%	39–78%
Bottom quintile	24–40%	12–24%

Only facilities with consistently collected interview data were included in the quintile calculations

Adherence rate is the cumulative rate of a video ever being offered to a particular long-stay patient or family member

Health-care system 1 had *N* = 22 facilities (11 top and 11 bottom) and health-care system 2 had *N* = 6 facilities (3 top and 3 bottom)

A total of 33 champion interviews were conducted and analyzed from among these 28 facilities (5 facilities had two champions). The champions were female (33/33) and mostly social workers (29/33). The professions of the four additional champions fell within administrative roles (*N* = 2) or nursing roles (*N* = 2).

Qualitative themes were encountered for all six CFIF domains, lending credence to the conceptual framework as the basis for exploring the experiences of the champions. Table 2 organizes the themes within the domains across three columns according to the classification: (1) paired-convergent, (2) paired-divergent, and (3) unpaired.

**Table 2:** Paired and unpaired themes across facility adherence status by Conceptual Framework of Implementation Fidelity domains

Modified Conceptual Framework of Implementation Fidelity domain	Paired-convergent	Paired-divergent	Unpaired
Intervention Complexity	• Intervention was simply designed (high and low)		
Participant Responsiveness	• Patients and families often changed advance directives after viewing the video (high and low)	• Patients and families were open (high) vs. reluctant (low) to view the video	• ACP video program is useful only on an as-needed basis (low) • Champions have personal investment in ACP (high)
Recruitment		• Champion’s efforts to recruit patients and families were strategic (high) vs. tentative (low)	
Quality of Delivery			• Champions actively combined showing the video with an ACP conversation (high)
Context			• Facilities had resource challenges (low) • Facilities had pre-existing ACP processes (low) • Facilities challenged by their ACP processes would benefit from the ACP video program (high)
Strategies to Facilitate Implementation		• Champions felt confident in their role (high) vs. not (low)	• Research team facilitation was helpful (high)

Facility adherence status: high = falls within top quintile for adherence within the health-care system; low = falls within bottom quintile for adherence within the health-care system

Conceptual Framework of Implementation Fidelity domains are from Hasson et al. (2010)

Paired-convergent indicates that the theme was represented in both high- and low-adherence facilities with similar findings. Paired-divergent indicates that the theme was represented in both high- and low-adherence facilities with dissimilar findings. Unpaired indicates that the theme was represented in only high- or only low-adherence facilities

### Intervention Complexity

There was no sharp distinction amongst champions from high- versus low-adherence facilities in their descriptions of the presentation and design of the PROVEN videos. One paired-convergent theme emerged. The overwhelming majority of champions in both high- and low-adherence facilities stated that the intervention and implementation were simply designed. A high-adherence facility champion related:

But [the ACP Video Program] made things easier. It’s easier to get triggered. It’s easier to do. It’s easier to track after. I mean, it’s a no-brainer. You know what I mean? You took away the negative of a process. [Participant 1]

A low-adherence facility champion expressed a similar perception:

[The videos are] easy to use. You just bring the tablet and you set it up… So, I think that it’s a smooth system. It’s easy to use and it’s clear. [Participant 2]

### Participant Responsiveness

A few themes (one paired-divergent, one paired-convergent, and two unpaired) emerged regarding PROVEN’s patients’, family members’, and champions’ reactions and attitudes towards the intervention. Patients’ and family members’ responsiveness, as perceived by the champions, tended to differ by facility adherence status, representing a paired-divergent theme. Champions in high-adherence facilities frequently described an openness on the part of patients and families to participate in the ACP Video Program. For example, one high-adherence facility’s champion noted:

[Family members are] pretty receptive. You know, they’re willing to listen, to be educated. Most of them will accept the [on-line access] link. [Participant 3]

In contrast, champions from low-adherence facilities often described patients and families as reluctant to view the videos. A low-adherence facility champion described this phenomenon:

It makes [the ACP conversation] harder, to be honest with you... After a while, the conversation becomes negative because you’re getting so much pushback from the family or from the resident that don’t want to use it. They don’t want to watch it.

[Participant 4]

Despite this contrast in responsiveness, champions from both high- and low-adherence facilities felt that patients and family members who did watch the video ended up reacting positively to it. In this paired-convergent theme, both types of facilities had champions who reported that a video sometimes motivated a change in advance directives, e.g., from full code to comfort care.

Champions’ own responsiveness to the ACP video program varied by facility adherence status. Facilities with low adherence tended to consider the intervention as needed on a conditional basis, an unpaired theme. One low-adherence facility champion stated:

It’s a great program. It’s a great resource to have. I think everybody should have the resource, but more so as an option, not necessarily a requirement… Let us use it as a resource as needed or as we feel it fit. [Participant 5]

On a few occasions, champions from high-adherence facilities portrayed a personal investment in ACP, which informed their positivity to the ACP video program, another unpaired theme. One such champion explained:

When I read [the principal investigator’s] book and looking at this process, it fills my heart with joy that he has taken this under his wing… It’s not only had a professional impact, but a personal impact. [Participant 6]

### Recruitment

A Recruitment paired-divergent theme related to how PROVEN’s champions solicited patients and family members to watch a video. High- versus low-adherence facilities differed in the comfort level or attitude with which they approached this task. High-adherence facilities had some champions who approached recruitment of nursing home residents and family members as a strategic effort, as one such champion explained:

Just by showing, presenting it to the patient as what it can do, what’s in it for them. With all sales, you know, we’re kind of selling this video, and with any sales, people want to know what’s in it for them. [Participant 7]

Some champions from low-adherence facilities approached recruitment with a more tentative stance, as expressed in the following quote:

It’s hard to walk up and say: “Well, it’s your time. You have to see this video again.” We don’t say that, obviously, but that’s how I feel about it. “Oh shoot. I’ve got to call this family in.” Or I’ve got to go approach them and say watch this thing again. [Participant 8]

### Quality of Delivery

One unpaired theme reflected the intent of PROVEN’s ACP video program to be an adjunct to general ACP discussions between champions and patients and families. High-adherence facility champions tended to use this dovetailed approach. A high-adherence facility champion referred to this tendency:

I usually try to have a discussion after it’s over and ask them if they have any questions and then within two days call them back after they’ve seen it to see if there was any questions, how they’re feeling, you know just to make sure that everything’s on an even kilter. [Participant 9]

### Context

CFIF’s Context construct emerged as three unpaired emergent themes that highlighted how intervention facilities’ characteristics and concurrent events (i.e., co-existing ACP interventions) differed by adherence status. Resource challenges in low-adherence facilities were cited by champions*.* This was epitomized by a low-adherence facility champion’s statement:

It’s no challenge as far as showing the video, but it’s hard for me because originally they sent four of us up there to train and it’s been pretty much me that’s been having to do that and being a social worker, I have a lot of duties and I just have to make the time for it and so I really need some help or maybe to get maybe our new unit managers on board if we could get them some training. [Participant 10]

Champions from low-adherence facilities also described less of a need for the ACP video program given the co-occurring ACP interventions locally. A champion from this type of facility explained:

Like I said, we are part of a big push anyway with our feed hospital as far as POLSTs and advance care planning as it is. We really deferred a lot mostly to the POLST form, have [the patients and families] review it, the docs going over it with them, and there just isn’t usually that need for the video. [Participant 5]

In a few high-adherence facilities, champions felt that new staff or facilities having a hard time with ACP would benefit the most from the ACP video program. When asked if she would recommend the ACP video program to other skilled nursing facilities, a high-adherence facility’s champion responded:

It’s definitely case by case… If the facility is having a lot of challenges with conversations with families and not having advance directives in place and things just kind of hanging out there, I think [the ACP video program] would be really great. [Participant 11]

### Strategies to Facilitate Implementation

Two key themes emerged (one unpaired and one paired-divergent) related to the research efforts by the team, corporate leaders, and champions to optimize implementation. High-adherence facility champions often commented on the positive impact the collaborative team leadership had on their ability to maintain fidelity. For example, as one high-adherence facility champion mentioned:

Well, we got on with our little meetings we have with the doctor, Dr. X [a PROVEN principal investigator]… He would kinda give us insight how we could pursue, you know even the difficult families, you know, what maybe we could go this route, instead of that way. Maybe go around instead of forward…and it kinda gave us a little insight on where we are lacking, try to include everyone in the mix, instead of just the few that we see all the time. [Participant 12]

In a few instances, champions’ own efforts to facilitate implementation could be characterized as qualitatively different across facility adherence status. A high-adherence facility champion explained her own approach, which reflected ongoing purposeful efforts at implementation despite challenges:

So, I mean, I’m trying to put a lot of little processes in place to actually implement better. It’s just, you know, it’s baby steps. [Participant 1]

In contrast, a low-adherence facility champion related her own sense of uncertainty about how to address implementation challenges she faced:

I told [a health-care system leader] that we had had a lot of changeover and it was nobody but me and that I needed some help and so, but, I don’t, nothing’s happened yet. So, I don’t know what I need to do on my end. [Participant 10]

## Discussion

This mixed-methods analysis of the PROVEN trial data provides insights into the implementation experiences in a large nursing home-based pRCT evaluating an ACP video program. Only one of the CFIF domains, Intervention Complexity, did not yield divergent or unpaired themes by facility adherence status, while the remaining five domains did (i.e., Participant Responsiveness, Recruitment, Quality of Delivery, Context, and Strategies to Facilitate Implementation). In terms of Intervention Complexity, both types of sites seemed to find the intervention simply designed. Of the most notable contrasts by facility types, low-adherence facilities were characterized by: (1) perceived negative patient and family responsiveness to the ACP video program (Participant Responsiveness), (2) tentative champion recruitment efforts (Recruitment), and (3) limited organizational resources (Context), while high-adherence facilities were characterized by: (1) perceived greater patient and family willingness to engage in the ACP video program (Participant Responsiveness), (2) strategic approaches to recruitment (Recruitment), (3) champions’ use of the video to bolster ACP conversations (Quality of Delivery), and (4) champions’ appreciation of facilitation by the research and leadership team (Strategies to Facilitate Implementation). Three key lessons regarding fidelity efforts in pRCTs can be gleaned from these findings: the importance of flexible fidelity, reciprocal facilitation, and organizational readiness. As modified CFIF domains (i.e., moderating factors of fidelity outcomes) supported these three lessons in an overlapping manner, domain-specific findings will be embedded throughout the following discussion of each lesson.

This work underscores the importance of embracing flexible fidelity when conducting pRCTs. Flexible fidelity recognizes the need to deliver core elements of an intervention while allowing for the purposeful adaptation of non-essential intervention features (i.e., features not critical to achieving valid intervention effectiveness) [4, 30]. Such adaptability enables the context-sensitive delivery of the intervention, which, in turn, enables program sustainability [30].

The need for such adaptability at the individual and organizational levels is apparent from our and others’ findings, especially those findings related to Participant Responsiveness and Context. PROVEN’s champion interviews underscored how individual stakeholder needs (i.e., the needs of patients, family members, and champions) should be factored into implementation efforts. Such an approach could foster positive Participant Responsiveness in these stakeholders, which was at times lacking in PROVEN’s low-adherence facilities. Other trials (both pRCTs and traditional RCTs) have suggested this same need to adjust interventions mid-stream. Potential factors that influence fidelity include the emerging needs of both patients (e.g., language literacy [31], and patient life and clinical circumstances [4, 32]) and providers (e.g., prior skill and competency [4, 31]). Given the challenges in PROVEN’s low-adherence facilities associated with local resources, organizational needs related to Context also emerged as an important consideration for adaptation. The other trials mentioned also found that organizational resources (e.g., limited staff time) and routines had an impact on implementation fidelity [4, 31, 32]. Our findings, consistent with other works in the literature, thus support that investigators should expect the need for flexible fidelity and factor this need throughout the process of developing, testing, and implementing interventions [30].

Our findings for Participant Responsiveness, Recruitment, and Strategies to Facilitate Implementation provide another lesson for fidelity in pRCTs: the value of reciprocal facilitation. We define reciprocal facilitation here as the early and ongoing engagement of stakeholders in research design and, reciprocally, the ongoing support of the leadership team (i.e., collaborative support offered by researchers and corporate leaders) of champion implementation efforts through continuous qualitative feedback and coaching. The more negative Participant Responsiveness to the intervention by PROVEN participants in low-adherence facilities may highlight the need for engaging all stakeholders from the initial planning stages of pRCTs onward. Such a strategy could enhance stakeholder buy-in to the intervention, as other studies have found. A quantitative analysis of PROVEN discovered that a characteristic of facilities associated with adherence was champion engagement (i.e., the degree of attendance at ongoing coaching calls) [6]. An RCT implementing INTERACT, a multi-component quality improvement program with an ACP component, also argues for the involvement of a variety of stakeholders when designing an implementation [33]. Indeed, early and ongoing stakeholder engagement has been championed to strengthen efforts that appropriately balance fidelity and adaptation in pRCTs [34].

Facilitation in the opposing direction, from leadership team to champion, can also play a critical role in fidelity for pRCTs by addressing issues relating to Participant Responsiveness, Recruitment, and Strategies to Facilitate Implementation. We found that in PROVEN’s high-adherence facilities, champions perceived that facilitation driven by the leadership team (i.e., ongoing qualitative feedback and coaching via conference calls) was beneficial. These leadership team-driven activities, promoted by other studies of palliative care interventions [35–38], may have differentially enhanced participant responsiveness if high-adherence (versus low-adherence) facility champions self-selected into conference call participation. Indeed, investigators from the INTERACT trial and from a feasibility study of another long-term care-based ACP intervention revealed the influence of sharing successes with champions [33, 39]. The studies found that positive results from program implementation reinforced providers’ motivation to maintain their implementation efforts. Furthermore, PROVEN champions approached recruitment more strategically in high- versus low-adherence facilities. Ongoing feedback and coaching can offer opportunities for champions to brainstorm with the leadership team and other champions on how to hone recruitment skills, especially if there is patient resistance to participation. Accordingly, providers in a pRCT of cognitive-behavioral therapies noted the need for training in how to adjust to patients’ circumstances during the implementation [4]. The overriding lesson is that leadership team-driven facilitation should be considered an active and core ingredient to successful implementation [32, 40].

In line with other studies, PROVEN’s findings (i.e., related to Recruitment and Context) highlight another lesson about fidelity in pRCTs: the need to assess organizational and champion readiness and to adapt accordingly. Tools such as the Organizational Readiness for Change Assessment (ORCA) and the Organizational Readiness for Implementing Change measure can assess the potential that an organization has for implementation success [41, 42]. Constructs measured within ORCA, for example, include contextual resources (e.g., limited staff time) and champion characteristics (e.g., underdeveloped recruitment skills), two areas of relative weakness in PROVEN’s low-adherence facilities. Our and others’ findings highlight the need for using such tools, not just initially, but also regularly thereafter (e.g., post-initial training [31]). With readiness information available, remediation plans can be implemented for low-adherence facilities and their champions. Such plans could include 1:1 champion meetings, observations of champion work flows, focused training on areas of weakness, and even replacement of champions if the preceding efforts did not work [38].

### Limitations

The limitations of this work should be noted. First, conducting qualitative interviews with patients and family members, in addition to champions, could have provided valuable triangulation of our findings. This was beyond the scope of this study, however. Second, we combined the two health-care systems’ data in the analysis despite their differing sizes and cultures. Third, our classification of some qualitative themes as unpaired could be misleading. One may not be able to draw inferences about the absence of a theme in one adherence type (e.g., low) that was present in the other adherence type (e.g., high) given that a number of issues could lead to this finding (e.g., interviewees seeking to answer in a socially desirable manner or inconsistencies by interviewers in asking questions across participants). We tried to counter this limitation by developing a detailed interview guide accompanied by interviewer-initiated prompts as needed. Finally, our findings may not be transferable to other long-term care settings, including nonprofit facilities.

## Conclusions

Findings from this large pRCT of a simple intervention suggest that flexible fidelity may be an important overarching principle for realizing optimal implementation in pRCTs. In this vein, reciprocal facilitation, i.e., champions aiding the leadership team in pRCT design and, conversely, the leadership team aiding champions with implementation, may dynamically enhance implementation fidelity. Facilities deemed at-risk of implementing pRCTs without fidelity could be identified by formally measuring organizational readiness early and throughout the implementation. Such facilities could then benefit from targeted remediation support.

## Supplementary information

**Additional file 1.** Champion Implementation Guide.

**Additional file 2.** Intervention Facility Implementation Guide.

**Additional file 3.** Interview guide 15 months post-implementation.

**Additional file 4.** Structured Analytic Codebook

## Data Availability

The data that support the findings of this study are available from the corresponding author (JAP) upon reasonable request.

## Abbreviations

ACP: Advance care planning
CFIF: Conceptual Framework of Implementation Fidelity
pRCT: Pragmatic randomized controlled trial
PROVEN: Pragmatic Trial of Video Education in Nursing Homes
RCT: Randomized controlled trial
