# Supplementary material for: Understanding implementation fidelity in a pragmatic randomized clinical trial in the nursing home setting:a mixed-methods examination
Source: Trials. 2019 Nov 28;20:656. doi: 10.1186/s13063-019-3725-5 (PMC6883560; doi:10.1186/s13063-019-3725-5)
Supplement: Supplementary file 2 — Additional file 2. Intervention Facility Implementation Guide. [file 13063_2019_3725_MOESM2_ESM.pdf]

Genesis HealthCare®

Advance Care Planning (ACP) Video Program

TOOLKIT A: USER GUIDE

Table of Contents

I. Introduction 4

II. The Advance Care Planning (ACP) Videos 5

III. Using The ACP Videos 7

A. When to show ACP videos 7

B. Choosing an ACP Video 7

C. Starting the Conversation 8

D. Showing a Video 9

E. Continuing the Conversation 9

F. Documenting and Translating Preferences into Advance Directives 10

IV. Getting Help 12

V. Appendices 13

THE PURPOSE OF THIS TOOLKIT:

1 To describe a suite of educational advance care planning videos that informs patients and families about their options for medical care in advanced illness.

2 To learn how to use these videos as part of the advance care planning conversations with your patients and their families.

I. Introduction

The Advance Care Planning (ACP) Video Program is an evidence-based intervention that provides simple and short videos to help empower patients in skilled nursing centers with and their families to meaningfully share in medical decision-making. It is important that patients with advanced illness receive medical care that is based on their values and beliefs. Including the videos in your conversations with patients will help them to make informed choices and find their voice so that they can share what matters most to them.

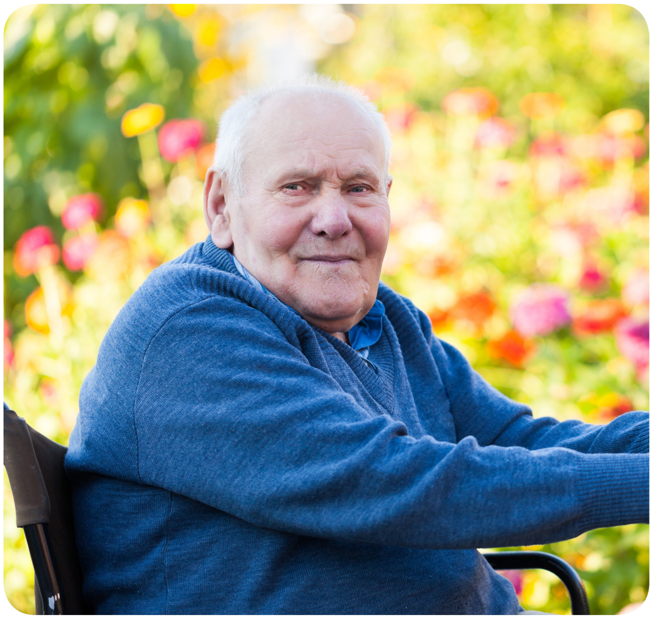

"Watching one of the videos about my choices for medical care helped me and my daughter really understand our options and make sure that I get the care that I want."

—Skilled nursing center patient

A skilled nursing center is the ideal place to use the ACP Video Program. Your patients are often very ill, elderly, and vulnerable. By sharing a video with them and having a conversation about ACP, you can help your patients and their families be at the center of and in control of their well-being.

"It's never easy making medical decisions for mom now that she has advanced dementia. But using one of the ACP videos allowed me to better understand what her doctor was asking me to think about. Now I get it."

—Daughter of a patient with advanced dementia

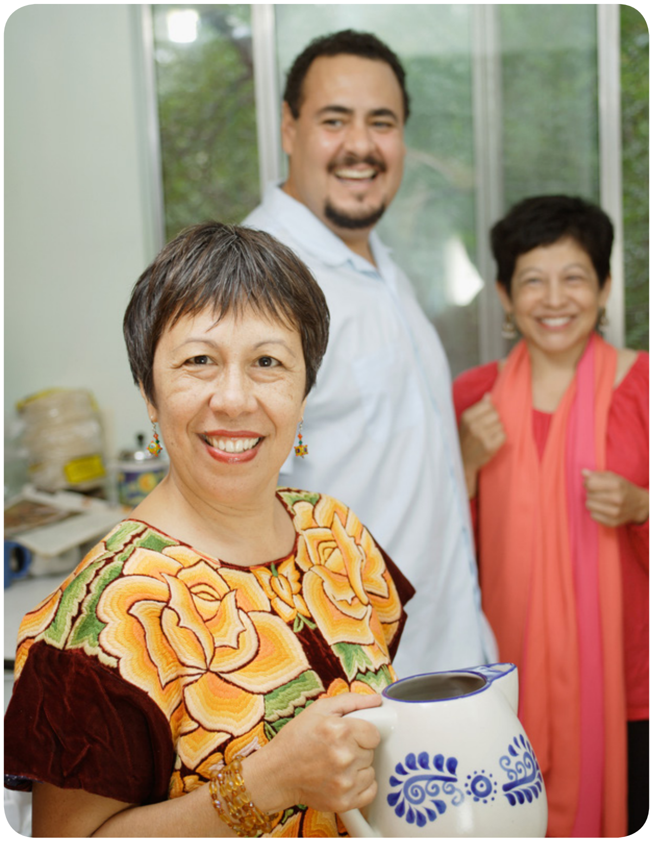

II. The Advance Care Planning (ACP) Videos

The ACP Videos include a suite of five videos covering major decisions that patients with advanced illness living in skilled nursing centers may need to make. The video titles are listed below, *(those marked with an asterisk are available in English and Spanish):*

- Goals of Care for Any Patient*
- Goals of Care for Patients with Advanced Dementia
- Decisions about Hospitalization*
- Decisions about Hospice*
- General Information about Advance Care Planning for Healthy Adults*

Except for Goals of Care: Advanced Dementia, which is meant for family members, the videos' narratives present options from the perspective of the patient as decision-maker. **There is a second version of the Goals of Care for Any Patient** video available which presents the same information at a slower rate of speech.

The five videos follow a similar structure that presents three general approaches for medical care available to patients in skilled nursing centers: Life-Prolonging Care; Limited Care; and Comfort Care, as shown in the table below. The program helps patients and their families to understand these three approaches, and determine which approach best fits with the patient's wishes for their medical care.

What are the Three Approaches to Medical Care?

Life Prolonging Care	Limited Medical Care	Comfort Care
• Goal to prolong life<br>• Wants all available treatments including: cardiopulmonary resuscitation (CPR), Mechanical Ventilation, and Intensive Care Unit (ICU)<br>• Care can only be provided in hospital	• Goal is to return to prior level of physical functioning before illness<br>• Treat reversible conditions<br>• May include hospitalization, intravenous fluids, antibiotics<br>• **NO** CPR, ICU, or Mechanical Ventilation<br>• Care may be provided in skilled nursing center or hospital	• Goal is to maximize comfort<br>• Only treatments to relieve suffering, such as oxygen and analgesics<br>• NO CPR, ICU, Mechanical Ventilation or intravenous fluids<br>• Usually cared for at skilled nursing center; hospitalize only if needed for comfort

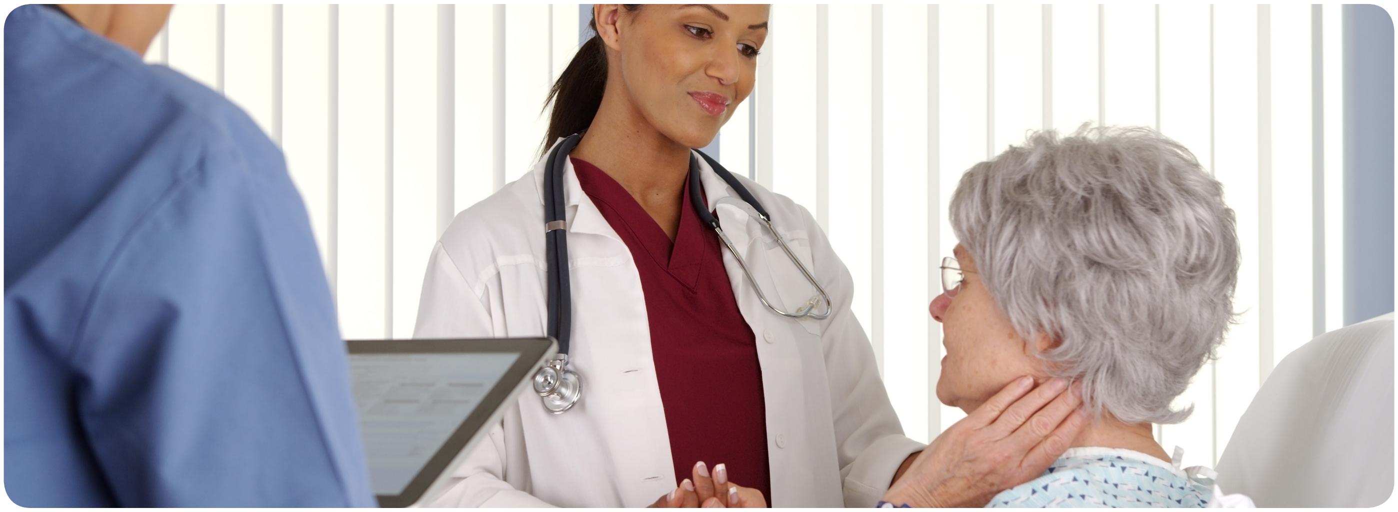

ACP Video Suite

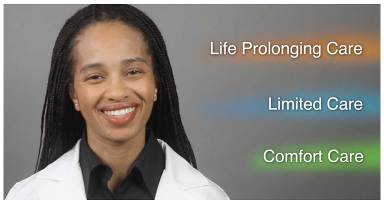

*Goals of Care for Any Patient**

This video helps patients understand and make decisions about their goals of care.

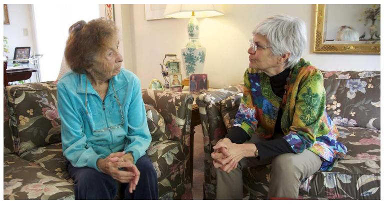

Goals of Care for Patients with Advanced Dementia

This video helps family members understand and make decisions for patients with advanced dementia.

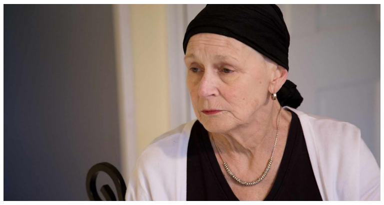

*Decisions about Hospice**

This video helps patients and their families understand and make decisions about hospice care.

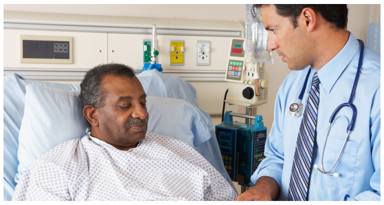

*Decisions about Hospitalization**

This video helps patients understand and make decisions about hospitalization.

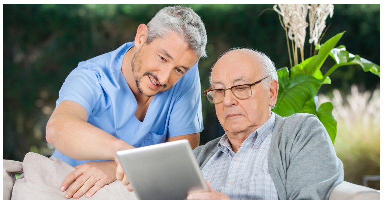

*General Information about Advance Care Planning for Healthy Adults**

This video helps generally healthy patients understand and make decisions about their long-term health goals.

***Spanish version available**

III. Using The ACP Videos

A. When to show ACP videos

There are key points in a patient's skilled nursing center stay that are ideal to show an ACP video to a patient and/or their family.

All newly admitted patients to the skilled nursing center are offered an ACP video **within one week of admission. Admission** is the optimal time to have an initial ACP conversation; the patient's clinical situation is being comprehensively reviewed by the health care team. Goals of care must be established with the patient and family.

All Patients are offered an ACP video **within one week of re-admission** to the skilled nursing center following a hospitalization.

All residents in the **long-term care setting** and/or their family members are offered ACP videos **every 6 months** so that they can revisit their goals of care and Advance Directives over time and with changes in health status. **This can be coordinated with regularly scheduled care planning meetings**. The **Hospitalization** and **Hospice** videos are shown to patients and families who are making decisions regarding those specific events.

Other times to offer an ACP video are when patients have a **major change in health status** (e.g., decline in cognition, new illness) or at **family meetings**. These are ideal opportunities to revisit goals of care and Advance Directives.

The table below presents the "Trigger Events" that will prompt a skilled nursing center care provider to offer an ACP video.

B. Choosing an ACP Video

Once you have identified the correct time to show a video, the next step is to choose the right video for the patient. For some patients, more than one video may be appropriate.

The most commonly used video is the **Goals of Care for Any Patient** video. It introduces broad choices for medical care, and is applicable to every patient with advanced illness in the nursing center. **The Goals of Care for Any Patient** video is a good choice to show all new admissions and at periodic reviews for long-term care residents. Show the **Goals of Care for Any Patient: Slower Pace** video to residents who are hearing-impaired, or whom, for any reason, may prefer to watch to the video at a slower pace. The only exception is for patients with advanced dementia.

The **Goals of Care for Patients with Advanced Dementia** video was specifically designed for the individual responsible for making decisions for patients with this condition (e.g., family members, health care decision-maker), and is shown to these individuals rather than the generic **Goals of Care for Any Patient** video.

Table 1. Events Triggering when an ACP Video is Offered

1)	ALL PATIENTS: Within One Week of Admission
2)	ALL PATIENTS: Within One Week of Re-admission from Hospital
3)	ALL PATIENTS: Significant Change in Health Care Status
4)	LONG-TERM CARE RESIDENTS: Every 6 months (Align with Scheduled Care Planning Meetings)
5)	FAMILY MEETINGS: About Goals of Care
6)	SPECIFIC DECISIONS: Covered by a Video (e.g., Hospice, Hospitalization)

All patients considering whether or not to be hospitalized in the event of an acute illness should be offered the **Hospitalization** video.

If hospice is available, the **Hospice** video is offered to patients who have an estimated prognosis of less than 6 months or whenever hospice is available.

The **Healthy Adults** video is to be used only for more robust patients admitted to the skilled nursing center for a short-stay to recover from a time-limited problem (e.g., rehabilitation after an elective knee replacement). These patients should be relatively well otherwise.

C. Starting the Conversation

The ACP videos are not a replacement for the conversation that clinicians and patients and families must have together about the options for medical care. The videos are meant to be just one component within this broader conversation. The videos enhance shared decision-making by providing a framework and visual images that help patients and families better understand their options. **The videos are a catalyst to having the conversation.**

Five key questions to ask to help start the ACP conversation are:

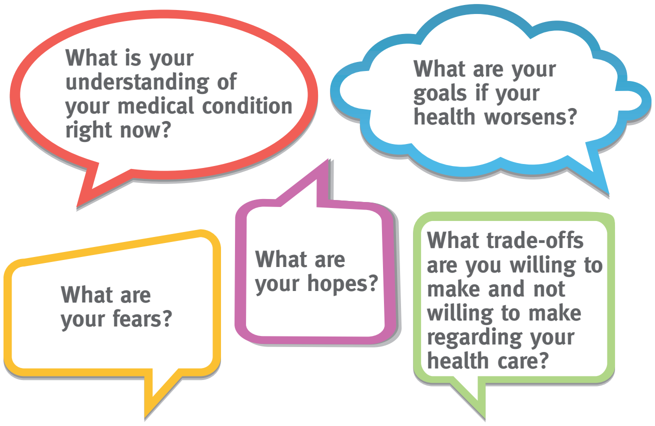

Be an active listener. Summarize what is being said as the conversation moves forward to ensure everyone is on the same page. Ask them to share their thoughts ("Please tell me more about that"), so that you can understand their perspective and how they are responding emotionally to their illness. Addressing emotional tension in the room makes it easier for the patient and his/her family to fully process the information that you are sharing with them about their choices for medical care.

Table 2. General Guidelines on Which Video to Show

PATIENT	VIDEO				
	Goals of Care	Advanced Dementia	Hospitalization	Hospice	Healthy Adults
Any Patient	✓		•	•	
Advanced Dementia		✓	•	•	
Any Patient Still Considering Hospitalization	•		✓	•	
6-month prognosis	✓		•	•	
Healthier patient with time-limited medical problem					✓

✓ Videos that are strongly suggested for these patients
• Videos that may also be appropriate for these patients

Be aware that the **cultural backgrounds**, religion, and spiritual beliefs of your patients may strongly influence their decisions regarding illness, advance care planning, and the end of life. Cultural sensitivity and respect for your patients' background is an essential component in helping them make decisions that honor their individual voice.

After starting the conversation, a good way to introduce the video is to say: *"I want to respect and honor your choices for medical care. I would like to watch an educational video with you that reviews your options for medical care. Many patients have found the video quite helpful. After we watch the video, we will continue our conversation."*

D. Showing a Video

There are several different ways to show a video. Your center will be provided with two tablet devices on which the videos are pre-programmed. The designated ACP Champions are the "keepers" of these tablets. The videos can also be viewed by visiting the weblink listed below and on the "Getting Help" page of this toolkit. This option is particularly useful to share the videos with family members who are unable to come to the skilled nursing center. We have provided you with cards that have this weblink to give to families who may want to watch the videos at home.

WEBLINK: http://bit.ly/ACPLibrary
Password: acplibrary1

Pick a quiet place; the patient and family should be able to watch the video in a non-rushed setting. When possible, you should try to watch the video with them. Patients and families may show particular interest in certain sections of the video. Take note of such moments and mention them after viewing the video *("I noticed that you found the Life-Prolonging Care part of the video particularly of interest…").*

While showing the video, note any non-verbal cues which can indicate **unease or discomfort**. The vast majority of people who have viewed these videos do so without difficulty. It can be normal for people to tear or become a little emotional when thinking about advance care planning. **However, if there is an unexpected, distressful reaction, stop the video, and immediately contact your supervisor and center ACP Champion.** They will make sure that a Serious Negative Reaction form is completed and sent to our staff within 24 hours of the event.

E. Continuing the Conversation

After the video is over, ask questions to help the patient and family focus their decision-making.

SOME HELPFUL QUESTIONS INCLUDE:

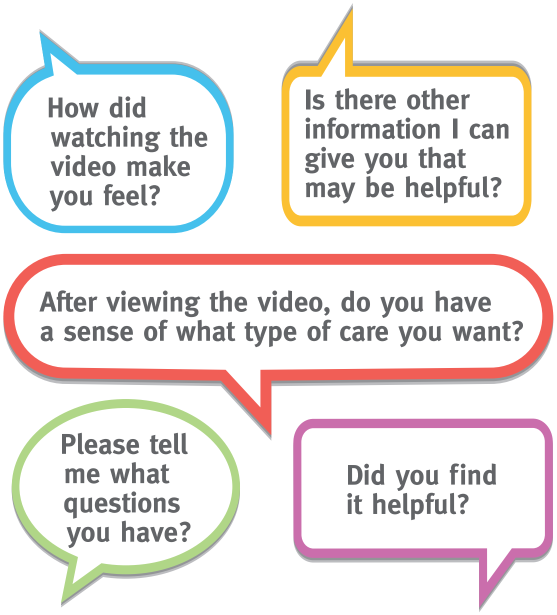

Exploring the feelings and viewing experience of the patient and family naturally leads to a conversation about the goals of care and Advance Directives.

The videos should serve as a springboard for these discussions. Patients may wish to defer decisions until they consult with others such as family members, friends, other clinicians, or chaplains. Reassure the

patient and his/her family that this is not a one-time conversation, but a process that takes place over time. Let them know that the nursing center staff is there to support them through this process.

Patients and families have questions too! In the following paragraphs are some frequently asked questions and suggested answers:

"Why is it important to make these decisions now?"

You are in a skilled nursing center. While we hope you stay as well as possible, there is always a chance your health will decline. Thinking about the type of care you want helps family members and health care providers make sure that you receive the medical treatments you desire.

"Can I discuss this with my family and friends?"

Absolutely! You should discuss your goals of care with your family and loved ones, or anyone else you think would be helpful. Family members are often asked to make decisions for you when you are very sick. So it is a good idea for them to know what you want.

"Can I change my choices?"

Yes. You can change your mind regarding your preferences at any point. Just let the health care professional providers taking care of you know.

It will likely take time and practice before you feel really comfortable having these conversations. After each conversation, take a few minutes and reflect on how the conversation went well and what could be improved. Below are some useful questions to ask yourself:

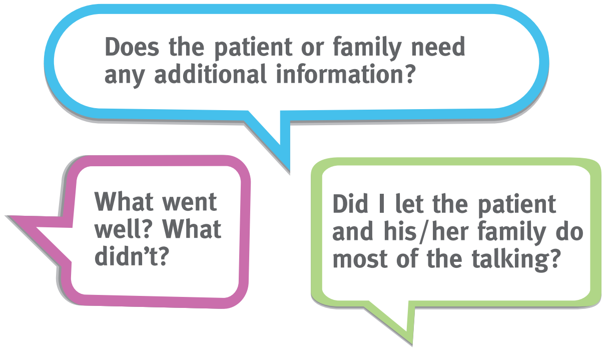

F. Documenting and Translating Preferences into Advance Directives

Timely and accurate documentation of ACP activities is critical to ensure that the patient's wishes are communicated to other health care providers.
There are two key pieces of documentation to attend to:

1. The Video Status Report User Defined Assessment (VSR UDA).

2. Review Advance Directives.

A. The VSR UDA is located in "Point Click Care", under the Clinical tab - Assessments. It must be completed each time a video is offered. **Even if the video is offered and not shown, for whatever reason, the VSR UDA must be completed.** The VSR UDA includes information on who viewed the video, which video was offered and who offered the video. There are also a couple of questions about the patients' reactions to the videos. Completion of the VSR UDA allows your skilled nursing center to track whether the videos are being offered on a routine basis at the appropriate times.

B. Ideally, the patient's preferences should be translated into Advance Directives that reflect his/her wishes. **Advance Directives** are medical orders; examples include:

- **Do-Not-Resuscitate (DNR)**
- **Do-Not-Intubate (DNI)**
- **Do-Not-Hospitalize (DNH)**
- **No feeding tubes**
- **No antibiotics**
- **No intravenous treatment**
- **Comfort measures only**

A clinician who is permitted to write medical orders, such as a physician, nurse practitioner, or physician's assistant, will be needed to "close the loop" in translating patient preferences into Advance Directives.

Some patients or families might be ready to complete Advance Directives immediately after watching an ACP video. Others may want to take their time before coming to a decision.

Advance Directive orders (e.g., Do-Not-Resuscitate [DNR], Do-Not-Hospitalize [DNH]) are critical in the skilled nursing center setting but vary as to how they are documented in the medical record. In some centers they are a part of the medical orders, while others use the state specific documents such as the Physician Orders for Life-Sustaining Treatment (POLST) or Medical Orders for Life-Sustaining Treatment (MOLST) forms. The options for care presented in the ACP videos generally align with the categories of care presented in the POLST and MOLST forms. The important thing is to be familiar with the process for Advance Directive documentation in your state and center and use it.

REFER TO:
http://central.genesishcc.com/sites/Clinical/ResourceLibrary/HealthCareDecisionMaking/default.aspx

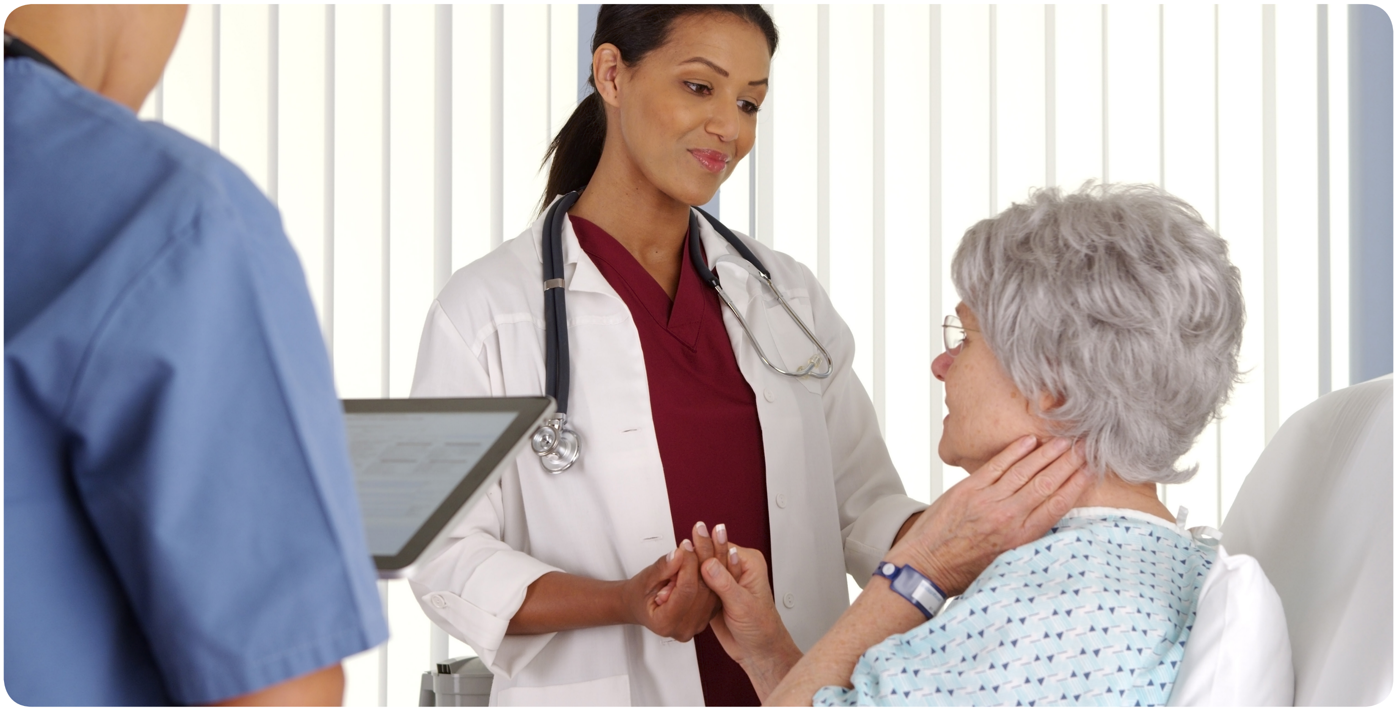

IV. Getting Help

Remember, you are not in this alone! Genesis Healthcare has invested in the resources necessary to help you and your staff provide the best care possible for your skilled nursing center's patients. Your regional clinical leadership, Genesis Healthcare contact, and the ACP Program team are always available to answer your questions. Additional training is always available upon request. **If you have any questions or need assistance, please contact:**

EAST DIVISION

Carol Eckerl

Area Specialist – Social Services
Carol.Eckerl@genesishcc.com
410-978-7274

Donna Moore

Sr. Specialist – Clinical Education
Donna.Moore-2@genesishcc.com
606-465-0328

WEST DIVISION

Tony Costa

Vice President Clinical Operations
Antonio.Costa@genesishcc.com
413-204-5128

Carolynne Adams

Sr. Director Quality
Carolynne.Adams@genesishcc.com
719-314-6208

You may also contact the ACP Project Staff and we will respond within one business day.

Phoebe Lehman

phoebelehman@hsl.harvard.edu
617-971-5313

Elaine Bergman

elainebergman@hsl.harvard.edu
617-971-5335

Weblink: **http://bit.ly/ACPLibrary**

Password: **acplibrary1**

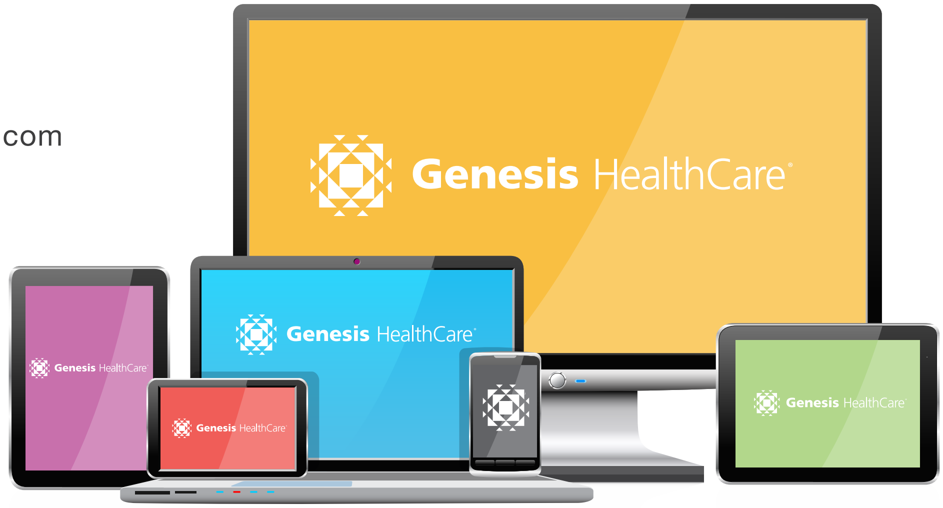

V. Appendices

Appendix A: Serious Negative Reaction Reporting

While very unlikely, serious distress by patients or family members in the intervention centers may occur as a result of watching a video. This type of reaction would be considered a Serious Negative Reaction and should be reported to Genesis Healthcare Center leadership and an ACP Program Contact. A Serious Negative Reaction may be manifested as a very negative emotional reaction while watching, or after watching, a video; asking for the video to be stopped; or leaving the room while a video is being shown. Due to the sensitive nature of the material, tearing up by the patient or proxy can be expected and is not deemed to be a reflection of distress.

Reporting and Follow-up Procedures:

If such a reaction occurs the following steps must be taken by ACP Champions and providers who show the videos (i.e., physician, nurse, social worker):

1. The video will be stopped.

2. The provider will report the reaction to his/her immediate supervisor and ACP Champions within 4 hours of occurrence. Together, these health professionals will determine the severity of the reaction.

A. **If deemed to be a true Serious Negative Reaction:**

i. The ACP Champion will complete a Serious Negative Reaction Form (see Appendix B), and submit it to the ACP Program Contacts listed on page 15 via email within 24 hours of the reaction.

ii. The provider or ACP Champion will also inform the ACP Program Contact within 24 hours of the Serious Negative Reaction by telephone.

3. The skilled nursing center provider and ACP Champion will check on the patient or family who experienced the distress at 6 and 24 hours after a Serious Negative Reaction to see how he/she is managing. If deemed necessary:

A. **The patient should be referred for counseling with a nursing center social worker or other mental health professional.**

B. **In the case of a family member, the nursing center provider may suggest to the proxy that he/she contact his/her own primary care provider.**

4. An ACP Program Contact will contact the Center ACP Champion within 48 hours of the Serious Negative Reaction to determine the status of the patient/family and whether further counseling was deemed necessary.

Appendix B: Serious Negative Reaction Form

CENTER ____ DATE OF FORM COMPLETION _____________________

ACP CHAMPION COMPLETING FORM: ____

The potential Serious Negative Reaction that could occur during use of the ACP videos is serious distress by patients or family members who viewed an ACP video. A manifestation of serious distress is considered to be: a very negative emotional reaction while watching, or after watching a video, asking for the video to be stopped, or leaving the room while a video is being shown. Due to the sensitive nature of the material, tearing up by the viewer can be expected and is not deemed to be a reflection of serious distress.

Date of Serious Negative Reaction ____________________ Time of Serious Negative Reaction ________:________AM/PM

A. Serious Negative Reaction observed by:
(Check all that apply)

- ☐ Direct Care Nurse
- ☐ Physician
- ☐ Nurse Practitioner
- ☐ Physician Assistant
- ☐ Social Worker
- ☐ Chaplain
- ☐ Director of Nursing
- ☐ Other _____________

B. Was Serious Negative Reaction observer an ACP Champion?

- ☐ No (Complete B1)
- ☐ Yes (Skip to C)

B1. Was Serious Negative Reaction reported to Advance Care Planning Champion within 4 hours of occurrence?

- ☐ No, Specify reason: ______________________________
- ☐ Yes, Specify ACP Champion:______________________

C. Who experienced the Serious Negative Reaction?

- ☐ Patient
- ☐ Family member
- ☐ Other (Please specify _____________________)

D. Which ACP video was shown? (Check all that apply)

- ☐ Goals of Care
- ☐ Advanced Dementia
- ☐ Hospitalization
- ☐ Hospice
- ☐ Healthy Patient

E. When did the Serious Negative Reaction happen?

- ☐ While watching video (Complete E1)
- ☐ After watching video

_______ minutes after watching video; skip to F

E1. Was the video stopped?

- ☐ No
- ☐ Yes

F. Please describe the Serious Negative Reaction below.

____

____

____

____

____

G. What intervention(s) were used to manage the Serious Negative Reaction?

____

____

____

____

____

H. What is the current status of the situation?

____

____

____

____

____

I. What follow-up actions are being taken with the person who experienced the Serious Negative Reaction?

____

____

____

____

____

Appendix B: Serious Negative Reaction Form (continued)

ACP CHAMPION: FOLLOW REPORTING INSTRUCTIONS BELOW:
Please call or email one of the following ACP Program Contacts within 24 hours of the Serious Negative Reaction:

PHOEBE LEHMAN
(617) 971-5313
phoebelehman@hsl.harvard.edu

ELAINE BERGMAN
(617) 971-5335
elainebergman@hsl.harvard.edu

1. Please indicate the person contacted *(Check all that apply)*

PHOEBE LEHMAN

Mode(s) of contact: *(Check all that apply)*

☐ Spoke with on telephone Date: ______________

☐ Left voicemail Date: ______________

☐ Emailed Date: ______________

ELAINE BERGMAN

Mode(s) of contact: *(Check all that apply)*

☐ Spoke with on telephone Date: ______________

☐ Left voicemail Date: ______________

☐ Emailed Date: ______________

Please email this form to an ACP Program Contact within 24 hours of Serious Negative Reaction.

2. Please indicate how, when and to whom the Serious Negative Reaction Form was sent *(Check all that apply)*

☐ **PHOEBE LEHMAN**

Serious Negative Reaction Form Sent via:

☐ Email Date: ______________

☐ Regular Mail (USPS) Date: ______________

Time: ________:________ AM/PM

☐ **ELAINE BERGMAN**

Serious Negative Reaction Form Sent via:

☐ Regular Mail (USPS) Date: ______________

☐ Emailed Date: ______________

Time: ________:________ AM/PM

Genesis HealthCare®

Adapted with permission from the Nous Foundation. © Copyright 2016 All rights reserved.
