# Supplementary material for: Understanding implementation fidelity in a pragmatic randomized clinical trial in the nursing home setting:a mixed-methods examination
Source: Trials. 2019 Nov 28;20:656. doi: 10.1186/s13063-019-3725-5 (PMC6883560; doi:10.1186/s13063-019-3725-5)
Supplement: Supplementary file 3 — Additional file 3. Interview guide 15 months post-implementation. [file 13063_2019_3725_MOESM3_ESM.pdf]

Genesis HealthCare®

Advance Care Planning (ACP) Video Program

TOOLKIT B

CENTER IMPLEMENTATION GUIDE

Contents

I. Implementing the ACP Video Program at Your Skilled Nursing Center 3

A. Introduction 3

B. Setting up the ACP Program 3

C. Planning Key Elements of Implementation 3

1. Who will show the videos to patients and their families? 3
2. When should the videos be offered? 3
3. How will the videos be shown? 4
4. How will the components of the ACP Video Program be documented? 4
5. What resources are available for training? 4
6. How you will evaluate the implementation of the ACP Video Program, and make adjustments based on your evaluation? 5

II. Getting Help 6

III. Appendices 7

I. Implementing the ACP Video Program at Your Skilled Nursing Center

A. Introduction

The purpose of this toolkit is to guide implementation of the ACP Video Program in the skilled nursing center.

This guide describes the essential components of the plan you need to have in place to set up the ACP Video Program.

B. Setting up the ACP Program

Introducing the ACP Video Program is a team effort. All interdisciplinary team members have an opportunity to contribute to the program's success. The staff members identified as the point people or "ACP Champions" at the skilled nursing center will lead the program. While not everyone at the center will actually show a video or have an ACP conversation, all providers who interact with patients and families, such as social workers, nurses, physicians, nurse practitioners, physician assistants, and clergy should be aware of and understand the program from the start.

C. Planning Key Elements of Implementation

The key elements listed below need to be clearly understood and in place before the program is implemented at your center.

1. **Who will introduce the videos to patients and families?**
2. **When will the videos be offered?**
3. **How will the videos be shown?**
4. **How will the components of ACP Video Program be documented?**
5. **What resources are available for training?**
6. **How you will evaluate the implementation of the ACP Video Program, and make adjustments based on your evaluation?**

1. Who will show the videos to patients and their families?

Each Genesis Healthcare center will designate at least two ACP Champions who will be responsible for offering and showing the videos. One of the ACP Champions should be the center's **Social Services Director**. Others can be a **social worker**, **Nurse Practitioner** or a **nurse**. Consider who is currently responsible for ACP planning at your center and what fits best with your workflow. Given that an ACP Video will be offered to **ALL patients upon admission**, it makes sense to select an individual who usually discusses goals of care and advance directives with new admissions.

2. When should the videos be offered?

There are key points in a patient's skilled nursing center stay that are ideal to show an ACP video to a patient and/or their family.

All newly admitted patients are offered an ACP video **within one week of admission. Admission is the optimal time to have an initial ACP conversation**; the patient's clinical situation is being comprehensively reviewed by the health care team and goals of care must be established with the patient and family.

All Patients are offered an ACP video within one week of re-admission to the skilled nursing center following a hospitalization.

All residents in the **long-term care setting** and/or their family members are offered ACP videos every 6 months so that they can revisit their goals of care and Advance Directives over time and with changes in health status. This can be coordinated with regularly scheduled care planning meetings.

The **Hospitalization and Hospice** videos are shown to patients and families who are making decisions regarding those specific events.

Other times to offer an ACP video are when patients

Table 1. Events Triggering when an ACP Video is Offered

1)	ALL PATIENTS: Within One Week of Admission
2)	ALL PATIENTS: Within One Week of Re-admission from Hospital
3)	ALL PATIENTS: Significant Change in Health Care Status
4)	LONG-TERM CARE RESIDENTS: Every 6 months (Align with Scheduled Care Planning Meetings)
5)	FAMILY MEETINGS: About Goals of Care
6)	SPECIFIC DECISIONS: Covered by a Video (e.g., Hospice, Hospitalization)

have a **major change in health status** (e.g., decline in cognition, new illness) or at **family meetings**. These are ideal times to revisit goals of care and Advance Directives.

3. How will the videos be shown?

There are several different ways to show a video. Your center will be provided with two tablet devices on which the videos are pre-programmed. The designated ACP Champions are the "keepers" of these tablets. The videos can also be viewed by visiting the weblink listed below and on the "Getting Help" page of this toolkit. This option is particularly useful to share the videos with family members who are unable to come to the skilled nursing center. We have provided you with cards that have this weblink to give to families who may want to watch the videos at home.

WEBLINK: http://bit.ly/ACPLibrary
PASSWORD: acplibrary1

4. How will the components of the ACP Video Program be documented?

There are two key pieces of documentation that are part of the ACP Video Program:

1. **The Video Status Report User Defined Assessment (VSR UDA)**
2. **Review Advance Directives**

The VSR UDA is integrated into Point Click Care on the Clinical tab under Assessments and must be completed each time a video is offered. During the planning stages, staff should be informed of the VSR UDA's location, how to access it, and when it must be completed. The VSR UDA must be completed each time a video is offered to a patient or his or her family, even if the video is not shown. An item on the form allows the staff member to indicate the reason why a video was not shown, if that is the case.

Advance Directive orders (e.g., DNR, DNH, etc.) are critical in the skilled nursing center setting but vary as to how they are documented in the medical record. In some centers, Advance Directives are part of the medical orders, while others use state specific forms such as the Physician Orders for Life-Sustaining Treatment (POLST) or Medical Orders for Life-Sustaining Treatment (MOLST) forms. As part of the planning phase for the ACP Video Program, ensure there is a standardized approach to Advance Directives documentation in your center. Viewing a video should prompt a review of the patient's Advance Directives, and the medical order on file should concur with the patient's wishes.

5. What resources are available for training?

There are two main components to ACP Program training. The first involves all the practical nuts and bolts of using the ACP Video Program, such as when to offer them, which video to use, how to use the electronic devices to show them and key elements of documentation. The second component involves learning how to integrate the videos into the broader ACP conversation.

The following summarizes some opportunities and resources ACP Champions have available for training and education:

- **Orientation session:** led by a Genesis Healthcare team member.
- **ACP Video Toolkit:** copies are available on-site, and also available online on GenesisU.
- **An educational module,** which covers information in the toolkits, is available for additional training on GenesisU.
- **Regular conference calls,** during which you and your colleagues at other nursing centers can call in to review actual cases with the project team. You can discuss what is going well with program implementation, as well as what needs to be improved. We encourage as many staff members to join these calls as possible.

6. How you will evaluate the implementation of the ACP Video Program, and make adjustments based on your evaluation?

The final planning element is how to evaluate and improve the ACP Video Program. There are several opportunities to do so. You should include the evaluation of the ACP Video Program as part of your regular QAPI (Quality Assurance and Performance Improvement) reviews.

On your own at the center, you can verify that all the patients admitted are being offered a video. This is easily done by using the center's EMR to check that a VSR UDA has been completed on each admission. Use a similar approach on your long-term care unit. Most long-term care patients should have a VSR UDA completed every six months.

Remember, **a VSR UDA MUST BE COMPLETED each time a video is offered.** If a video was NOT offered, check to see if the reason makes sense clinically (e.g., patient is already on hospice).

You will be sent several monthly reports from our partners. These reports include:

- A report summarizing the total number of VSR UDAs completed compared to the total number of admissions and readmissions at your center. These data come from the Point Click Care VSR UDA and census transactions.
- An benchmarking report generated by our partners at Brown University will be sent to you quarterly. This report will describe the proportion of new admissions with a completed VSR UDA in the prior 90 days, as well as long-term care residents with a VSR UDA completed in the prior 6 months in your center, compared to other Genesis centers. These data will come from the Point Click Care, VSR UDA and MDS reports.
- An additional benchmarking report generated by our partners at Brown University will be sent to you quarterly. This report will describe the proportion of new admissions with a VSR UDA completed in the prior 90 days, and long-term care residents with a VSR UDA completed in the prior 6 months in your center compared to other Genesis HealthCare centers. These data are generated from the AHT, VSR UDA, and MDS reports.

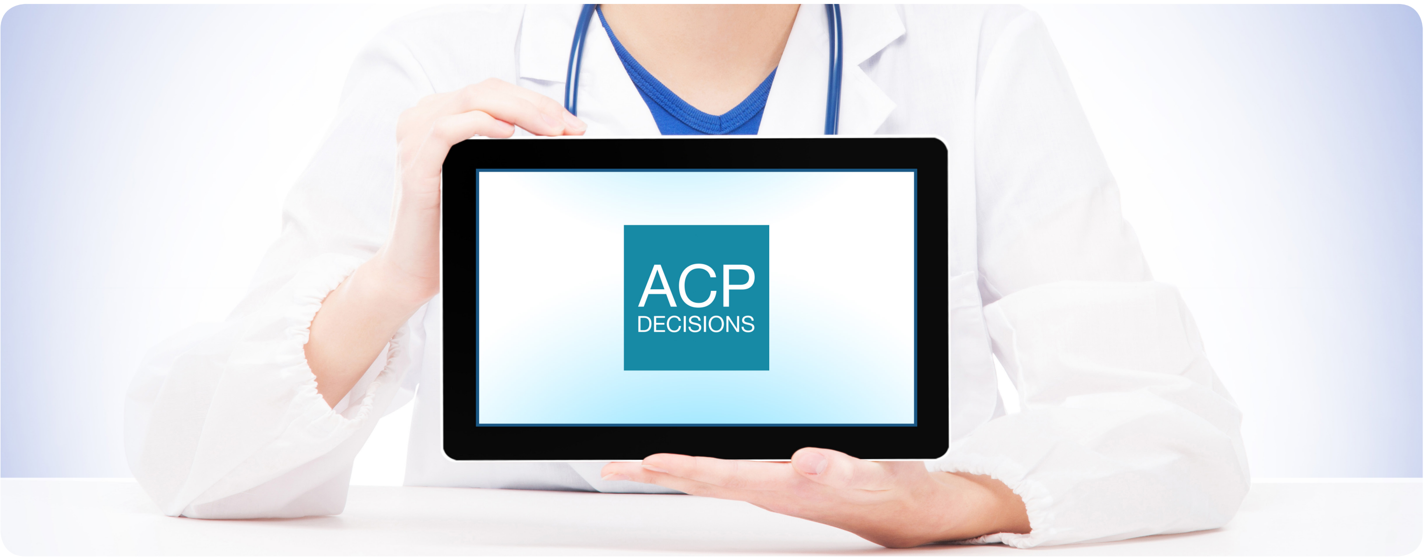

II. Getting Help

Remember, you are not in this alone! Genesis Healthcare has invested in the resources necessary to help you and your staff provide the best care possible for your skilled nursing center's patients. Your regional clinical leadership, Genesis Healthcare contact, and the ACP Program team are always available to answer your questions. Additional training is always available upon request. **If you have any questions or need assistance, please contact:**

EAST DIVISION

Carol Eckerl

Area Specialist – Social Services
Carol.Eckerl@genesishcc.com
410-978-7274

Donna Moore

Sr. Specialist – Clinical Education
Donna.Moore-2@genesishcc.com
606-465-0328

WEST DIVISION

Tony Costa

Vice President Clinical Operations
Antonio.Costa@genesishcc.com
413-204-5128

Carolynne Adams

Sr. Director Quality
Carolynne.Adams@genesishcc.com
719-314-6208

You may also contact the ACP Project Staff and we will respond within one business day.

Phoebe Lehman

phoebelehman@hsl.harvard.edu
617-971-5313

Elaine Bergman

elainebergman@hsl.harvard.edu
617-971-5335

TO ACCESS THE ACP VIDEOS, VISIT:

WEBLINK: http://bit.ly/ACPLibrary
PASSWORD: acplibrary1

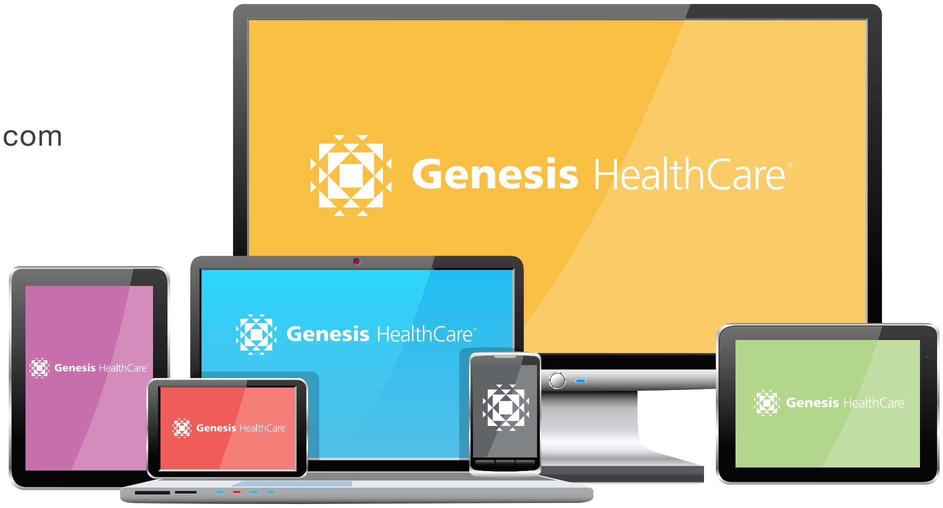

III. Appendices

Appendix A: Serious Negative Reaction Reporting

While very unlikely, serious distress by patients or family members in the intervention facilities may occur as a result of watching a video. This type of reaction would be considered a Serious Negative Reaction and should be reported to PruittHealth leadership and an ACP Program Contact. A Serious Negative Reaction may be manifested as a very negative emotional reaction while watching, or after watching, a video; asking for the video to be stopped; or leaving the room while a video is being shown. Due to the sensitive nature of the material, tearing up by the patient or proxy can be expected and is not deemed to be a reflection of distress.

Reporting and Follow-up Procedures:

If such a reaction occurs the following steps must be taken by ACP Champions and providers who show the videos (i.e., physician, nurse, social worker):

1. The video will be stopped.

2. The provider will report the reaction to his/her immediate supervisor and ACP Champions within 4 hours of occurrence. Together, these health professionals will determine the severity of the reaction.

   A. **If deemed to be a true Serious Negative Reaction:**

   i. **The ACP Champion will complete a Serious Negative Reaction Form (see Appendix B), and submit it to the ACP Program Contacts listed on page 9 via email within 24 hours of the reaction.**

   ii. **The provider or ACP Champion will also inform the ACP Program Contact within 24 hours of the Serious Negative Reaction by telephone.**

3. The nursing center provider and ACP Champion will check on the patient or family who experienced the distress at 6 and 24 hours after a Serious Negative Reaction to see how he/she is managing. If deemed necessary:

   A. **The patient should be referred for counseling with a nursing center social worker or other mental health professional.**

   B. **In the case of a family member, the nursing center provider may suggest to the proxy that he/she contact his/her own primary care provider.**

4. An ACP Program Contact will contact the facility ACP Champion within 48 hours of the Serious Negative Reaction to determine the status of the patient/family and whether further counseling was deemed necessary.

Appendix B: Serious Negative Reaction Form

CENTER ____ DATE OF FORM COMPLETION _______________________

ACP CHAMPION COMPLETING FORM ____

The potential Serious Negative Reaction that could occur during use of the ACP videos is serious distress by patients or family members who viewed an ACP video. A manifestation of serious distress is considered to be: a very negative emotional reaction while watching, or after watching a video, asking for the video to be stopped, or leaving the room while a video is being shown. Due to the sensitive nature of the material, tearing up by the viewer can be expected and is not deemed to be a reflection of serious distress.

Date of Serious Negative Reaction ____________________ Time of Serious Negative Reaction ________:________AM/PM

A. Serious Negative Reaction observed by:
(Check all that apply)

- ☐ Direct Care Nurse
- ☐ Physician
- ☐ Nurse Practitioner
- ☐ Physician Assistant
- ☐ Social Worker
- ☐ Chaplain
- ☐ Director of Nursing
- ☐ Other _____________

B. Was Serious Negative Reaction observer an ACP Champion?

- ☐ No (Complete B1)
- ☐ Yes (Skip to C)

B1. Was Serious Negative Reaction reported to Advance Care Planning Champion within 4 hours of occurrence?

- ☐ No, Specify reason: ______________________________
- ☐ Yes, Specify ACP Champion:______________________

C. Who experienced the Serious Negative Reaction?

- ☐ Patient
- ☐ Family member
- ☐ Other (Please specify _____________________)

D. Which ACP video was shown? (Check all that apply)

- ☐ Goals of Care
- ☐ Advanced Dementia
- ☐ Hospitalization
- ☐ Hospice
- ☐ Healthy Patient

E. When did the Serious Negative Reaction happen?

- ☐ While watching video (Complete E1)
- ☐ After watching video

_______ minutes after watching video; skip to F

E1. Was the video stopped?

- ☐ No
- ☐ Yes

F. Please describe the Serious Negative Reaction below.

____

____

____

____

____

G. What intervention(s) were used to manage the Serious Negative Reaction?

____

____

____

____

____

H. What is the current status of the situation?

____

____

____

____

____

I. What follow-up actions are being taken with the person who experienced the Serious Negative Reaction?

____

____

____

____

____

Appendix B: Serious Negative Reaction Form (continued)

ACP CHAMPION: FOLLOW REPORTING INSTRUCTIONS BELOW:
Please call or email one of the following ACP Program Contacts within 24 hours of the Serious Negative Reaction:

PHOEBE LEHMAN
(617) 971-5313
phoebelehman@hsl.harvard.edu

ELAINE BERGMAN
(617) 971-5335
elainebergman@hsl.harvard.edu

1. Please indicate the person contacted *(Check all that apply)*

PHOEBE LEHMAN

Mode(s) of contact: *(Check all that apply)*

- ☐ Spoke with on telephone Date: ______________
- ☐ Left voicemail Date: ______________
- ☐ Emailed Date: ______________

ELAINE BERGMAN

Mode(s) of contact: *(Check all that apply)*

- ☐ Spoke with on telephone Date: ______________
- ☐ Left voicemail Date: ______________
- ☐ Emailed Date: ______________

Please email this form to an ACP Program Contact within 24 hours of Serious Negative Reaction.

2. Please indicate how, when and to whom the Serious Negative Reaction Form was sent *(Check all that apply)*

☐ **PHOEBE LEHMAN**

Serious Negative Reaction Form Sent via:

- ☐ Email Date: ______________
- ☐ Regular Mail (USPS) Date: ______________

Time: ________:________ AM/PM

☐ **ELAINE BERGMAN**

Serious Negative Reaction Form Sent via:

- ☐ Regular Mail (USPS) Date: ______________
- ☐ Emailed Date: ______________

Time: ________:________ AM/PM

Adapted with permission from the Nous Foundation. © Copyright 2016 All rights reserved.
