# Supplementary material for: Understanding implementation fidelity in a pragmatic randomized clinical trial in the nursing home setting:a mixed-methods examination
Source: Trials. 2019 Nov 28;20:656. doi: 10.1186/s13063-019-3725-5 (PMC6883560; doi:10.1186/s13063-019-3725-5)
Supplement: Supplementary file 4 — Additional file 4. Structured Analytic Codebook [file 13063_2019_3725_MOESM4_ESM.pdf]

Domain	CFIF Definition*	Operational Definition
**Context**	"Importance of context for program implementation has been highlighted by several other authors. (Pettigrew, 1990; Pettigrew, 1992; Pettigrew, 1997; Steckler et al, 2002) Lipsey (2000) emphasized the importance of taking into account surrounding social systems, such as structures and cultures of organizations and groups, inter-organizational linkages, and historical as well as concurrent events, when assessing program implementation. Pettigrew and Whipp's (1990) model of strategic change management defined context together with content and process as main determinants of change."	Describes the culture around advance care planning prior to and including PROVEN's training or implementation processes. Includes descriptions of culture/resources/external mediators that affect implementation.
**Intervention Complexity**	"Intervention complexity has been found to influence the implementation fidelity, i.e., complex interventions were more difficult to implement with high fidelity than simple interventions. (Greenhalgh et al, 2004) Complexity refers to both description of the intervention and the real nature of the intervention. Interventions described in detail are more likely expected to be implemented with high fidelity than ones with vague descriptions."	Describes the video as being understandable or as clarifying concepts.
**Participant Responsiveness**	"Participant responsiveness refers both to individuals receiving the intervention and individuals responsible for delivering it. Higher levels of implementation fidelity are assumed to be achieved if those responsible for delivering an intervention are enthusiastic about it. Similarly, the uptake of the intervention depends on the responsiveness of those receiving it."	Describes the responsiveness (i.e., attitudes or reactions) to the intervention itself or to the research team's implementation activities. Includes responsiveness of the Champion, non-Champion staff, patients, and family members.
**Quality of Delivery**	"Quality of delivery concerns the appropriateness of the delivery process for achieving what was intended. Dusenbury et al. (2003) defined quality of delivery as the extent to which a provider approaches a theoretical ideal in terms of delivering program content.'"	Describes the manner in which the video was shown, regardless of whether the video viewing occurred in person or via on-line link. Two important distinctions: 1) this domain relates to program-level delivery not the meta-level sense of implementation amongst Champions, non-Champion staff, facility leadership, and the research team (which is what the "strategies to facilitate implementation" domain pertains to), 2) this domain stands in contrast to the "recruitment" domain which is about all processes leading up to but excepting the approach to showing the video).
**Recruitment**	"Recruitment refers to procedures that were used to attract potential program participants. Baranowski and Stables (2000) argued that recruitment was a key propro cess evaluation component. Some of the aspects to be evaluated were suggested to be reasons for nonparticipation among potential participants, subgroups that were less likely to participate, and consistency of recruitment procedures among potential participants."	Describes all processes beginning prior to offering the video, and proceeding through the offer up until the point of showing the video. An important distinction: this domain stands in contrast to the "quality of delivery" domain which refers to the process of showing the video.
**Strategies to Facilitate Implementation**	"Facilitation strategies such as provision of manuals, guidelines, training, and feedback, may be used both to optimize and to standardize implementation fidelity. However, more facilitation strategies do not necessarily mean better implementation. Instead facilitation might be highly dependent on the complexity of the intervention."	Describes any strategies used by the research team or the Champions/non-Champion staff/facility leadership to promote efforts at offering the videos. An important distinction: this domain relates to the meta-level sense of implementation amongst Champions, non-Champion staff, facility leadership, and the research team not program delivery from the Champion to the end-user (which is what the "quality of delivery" domain pertains to).
***Verbatim from Hasson (2010)**		
